# Desolvation Processes in Channel Solvates of Niclosamide

**DOI:** 10.1021/acs.molpharmaceut.3c00481

**Published:** 2023-10-18

**Authors:** Jen E. Mann, Renee Gao, Shae S. London, Jennifer A. Swift

**Affiliations:** † Department of Chemistry, Georgetown University, 37th and O Streets NW, Washington, District of Columbia 20057-1227, United States

**Keywords:** niclosamide, solvate, synchrotron diffraction, mechanism, desolvation

## Abstract

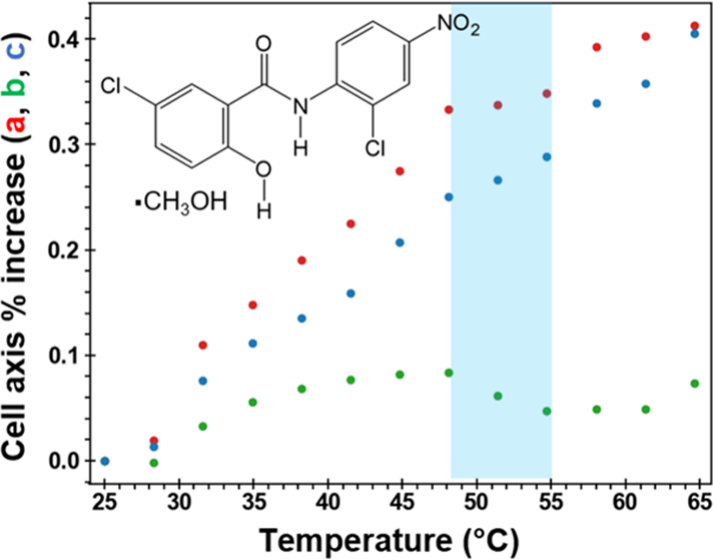

The antiparasitic drug niclosamide (NCL) is notable for its ability to crystallize in multiple 1:1 channel solvate forms, none of which are isostructural. Here, using a combination of time-resolved synchrotron powder X-ray diffraction and thermogravimetry, the process-induced desolvation mechanisms of methanol and acetonitrile solvates are investigated. Structural changes in both solvates follow a complicated molecular-level trajectory characterized by a sudden shift in lattice parameters several degrees below the temperature where the desolvated phase first appears. Model fitting of kinetic data obtained under isothermal heating conditions suggests that the desolvation is rate-limited by the nucleation of the solvent-free product. The desolvation pathways identified in these systems stand in contrast to previous investigations of the NCL channel hydrate, where water loss by diffusion initially yields an anhydrous isomorph that converts to the thermodynamic polymorph at significantly higher temperatures. Taking the view that each solvate lattice is a unique “pre-organized” precursor, a comparison of the pathways from different starting topologies to the same final product provides the opportunity to reevaluate assumptions of how various factors (e.g., solvent binding strength, density) influence solid-state desolvation processes.

## Introduction

Many active pharmaceutical ingredients (APIs) can crystallize as solvates.^1−7^ Though the number of APIs marketed as solvates (other than hydrates) is relatively small,^8,9^ solvates can play an important role in the drug development process. When viewed as preassembled “precursor” phases, intentional desolvation can yield solvent-free forms that may or may not be otherwise attainable by direct solution growth methods. The products of solid-state desolvation reactions are hard to predict a priori, but for any given reaction, there are at least three potential general outcomes. Upon solvent loss, the lattice may be retained resulting in a lower-density isomorphous desolvate,^10^ rearrange to a crystalline structure with a different three-dimensional lattice, or collapse to an amorphous phase. Desolvation may yield one (or more) metastable phase that could undergo further transformations to the final product. In the absence of time-resolved structural studies, intermediate phases can go undetected, complicating efforts to understand the solid-state transformation mechanism on the molecular level. To realize the full potential of solid-state desolvation reactions as a means to generate novel polymorphs, insight that establishes the molecular-level pathway between the solvate and solvent-free forms is needed.

**Figure 1:**
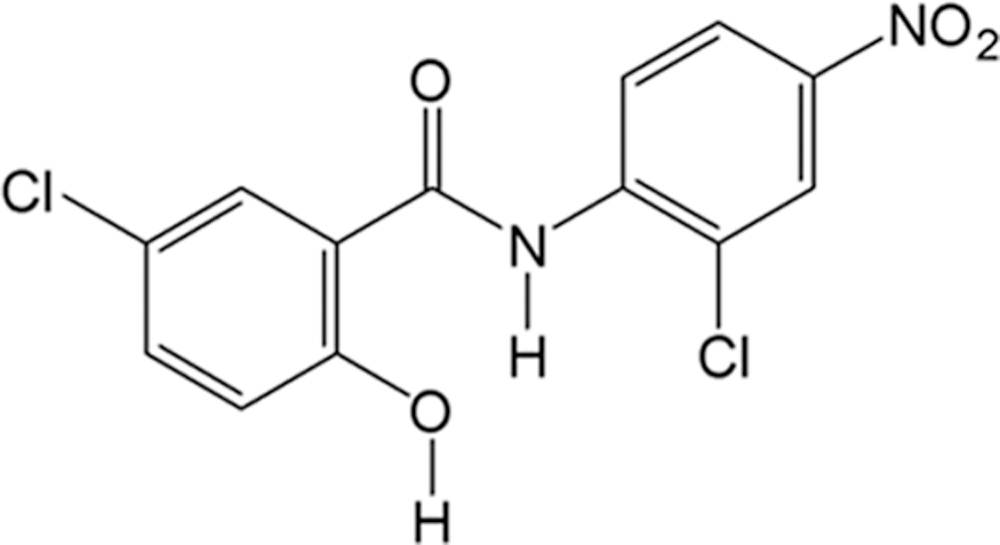
Molecular structure of niclosamide (NCL).

Niclosamide (NCL, Figure 1) is designated by the WHO as an Essential Medicine.^11^ Used as an intestinal anthelmintic for the past half-century, several more recent studies have sought to repurpose NCL as an antiviral,^12−15^ antibacterial,^16,17^ or cancer^18−20^ therapeutic. Its poor aqueous solubility has prompted efforts to identify other crystalline forms^21−27^ including two single-component polymorphs, two monohydrates (H_A_ and H_B_), and multiple solvates. Previous reports indicate that some of these solid forms can interconvert. For example, acetone and tetrahydrofuran solvates rapidly lose solvent when removed from the mother liquor.^21,23^ Our own analysis of both H_A_ and H_B_ revealed that process-induced dehydration (at RH = 0%) yielded anhydrate Form 1 (F1) as the final product but via distinctly different pathways.^28^

Comparative studies of compositionally different precursor phases of the same API can provide valuable insight into how structural factors affect the mechanisms, kinetics, and products of desolvation reactions. Like H_A_, in NCL monosolvates with methanol (S_MeOH_),^24^ acetonitrile (S_ACN_),^21^ and THF (S_THF_),^23^ the solvent molecules occupy one-dimensional channels. However, neither of the two forms are isostructural. Using a combination of time-resolved synchrotron powder X-ray diffraction (SPXRD) and thermogravimetric (TGA) kinetic studies, here we report on the process-induced desolvation of S_MeOH_ and S_ACN_ under the same conditions (RH = 0%) as the previous hydrate study. Though desolvation of H_A_ occurs via diffusion, here we show that S_MeOH_ and S_ACN_ can lose some fraction of solvent while retaining the same lattice; each ultimately follows an alternate mechanistic pathway to access the same final solvent-free product.

## Experimental Methods

### Materials

Niclosamide (NCL) was purchased from Sigma-Aldrich (≥98%) and used as received. Acetonitrile and methanol were obtained from EMD Chemical and Fisher Scientific. Both solvents were reagent grade or higher.

#### Preparation of NCL Solvates

##### Methanol Solvate (S_MeOH_)

S_MeOH_ was prepared by refluxing NCL (400 mg) in 20 mL of methanol for 30 min.^29^ The growth solution was cooled to room temperature before being transferred to glass vials that were capped and sealed with parafilm. S_MeOH_ grows as clusters of stubby yellow needles, with lengths typically <100 μm.

^1^H NMR (S_MeOH_ dissolved in CDCl_3_) δ: 11.20 (1H, s), 8.75 (1H, m), 8.39 (1H, s), 8.24 (1H, d), 7.55 (1H, s), 7.46 (1H, d), 7.02 (1H, m), 3.50 (3H, s).

##### Acetonitrile Solvate (S_ACN_)

S_ACN_ was prepared by recrystallization from acetonitrile.^21^ NCL (60 mg) was dissolved in 20 mL of acetonitrile and heated in a hot water bath with stirring. Upon cooling to room temperature, the growth solution was transferred to glass vials. Crystals of S_ACN_ appear immediately as colorless needles with lengths that can reach 200 μm. ^1^H NMR (S_ACN_ dissolved in CDCl_3_) δ 11.20 (1H, s), 8.73 (1H, m), 8.39 (1H, s), 8.25 (1H, d), 7.55 (1H, s), 7.44 (1H, d), 7.04 (1H, m), 2.01 (3H, s).

Micrographs of solvate crystals were collected on an Olympus BX-50 polarizing microscope fitted with a Lumenera Xfinity 2.0 camera (Figure S1).

##### Monohydrate A (H_A_)

H_A_ was obtained using the procedure described in ref (28).

##### Thermal Analysis

A TA Instruments Discovery DSC 25 and a TA Instruments SDT_Q500 simultaneous TGA-DSC analyzer were used to determine the desolvation temperature and corresponding weight loss of each solvate type. For DSC, phase-pure samples (2–5 mg) were lightly ground in a mortar and pestle, placed in covered aluminum pans with unsealed lids, and heated at 5 °C/min from room temperature to 250 °C. Unground samples were also tested. For TGA, lightly ground samples (2–5 mg) were placed in open ceramic pans and heated at a rate of 5 °C/min to a maximum temperature of 120 °C. All reported transition temperatures and weight losses are an average of at least triplicate measurements. The calculated solvent content in H_A_, S_MeOH_, and S_ACN_ are 5.22, 8.91, and 11.14 wt %, respectively.

##### Solid-State Dehydration Kinetics

TGA kinetic data were collected on lightly ground S_ACN_ and S_MeOH_ maintained at 40, 45, and 50 °C. Solid-state kinetic analyses were based on triplicate measurements at each isothermal temperature. The fraction desolvated (α) was determined from the percent weight loss over time relative to the total measured weight change. Model-based^30−32^ (Table S1) and model-free^33−36^ analyses utilized data in the linear regions of the TGA curves (0.1 < α < 0.9). The activation energy (*E*_a_) associated with desolvation was determined from Arrhenius plots. Each reaction model was assessed in terms of the correlation coefficient (*R*^2^) to determine the model(s) with the best fit. Changes in the *E*_a_ over the reaction time period were assessed by using model-free methods.

##### Powder X-ray Diffraction (PXRD)

In order to confirm the phase purity of all materials, PXRD data were collected at room temperature on lightly ground samples using a Rigaku Ultima IV diffractometer (Cu Kα radiation, 40 kV tube voltage, 30 mA current). Data from 2θ = 3–40° were collected with a scan speed of 2.0°/min and compared against simulated powder patterns from CIF files available in the CSD: S_ACN_ (refcode: KUKROZ^21^), S_MeOH_ (GOJLIC^24^), H_A_ (OBEQAN01^21^), and anhydrate F1 (HEBFUR^22^).

##### Time-Resolved Synchrotron Powder X-ray Diffraction (sPXRD)

Time-resolved synchrotron PXRD desolvation data on S_MeOH_ and S_ACN_ were collected on beamline 17-BM-B at the Advanced Photon Source (APS). The beamline is equipped with a Si(311) monochromator, a PerkinElmer a-Si Flat Panel PE1621 area detector, and an Oxford Cryosystems Cryostream 700+. All data were generated with a λ = 0.24087–0.45390 Å (51.5–27.3 keV). In variable-temperature experiments (heating rate = 10 °C/min, max. temperature 115 °C), and in isothermal experiments at 40, 45, and 50 °C, S_ACN_ and S_MeOH_ were hand-ground in a small amount of growth solution and then loaded into Kapton or quartz capillaries (OD = 1.1 mm) and stoppered with glass wool at each end. Capillaries were mounted in a flow cell^37^ maintained under a dry He atmosphere (5 mL/min) and continuously rocked at 10–15°. In situ data were collected with an exposure time of 2.0 s and summed over 10 images, allowing a high-Q-range sPXRD pattern to be collected every ∼20 s. Image processing and integration were done in GSAS-II^38^ and Pawley pattern refinement in TOPAS-V6.^39^

## Results and Discussion

**Figure 2:**
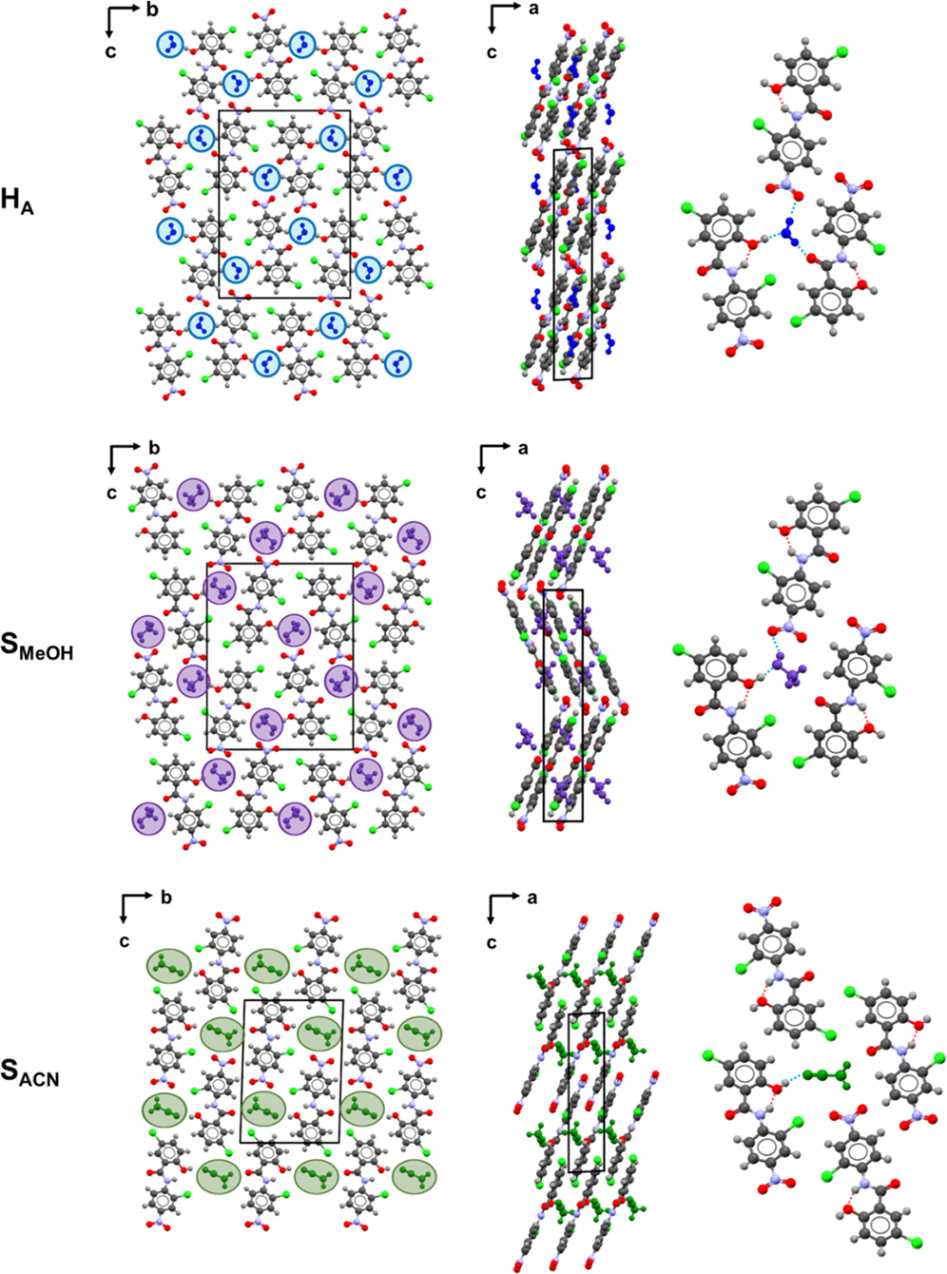
Packing diagrams of H_A_, S_MeOH_, and S_ACN_. NCL molecules form face-to-face π-stacks, creating one-dimensional channels occupied by solvent molecules. Each structure is viewed (left) down the *a*-axis, the π-stacking and channel direction, and (middle) down the *b*-axis with the channel direction horizontal. NCL molecules are colored by atom type, and water (blue), methanol (purple), and acetonitrile (green). (right) Close-up view of the NCL···solvent hydrogen-bonding interactions.

The structures of channel solvates H_A_, S_MeOH_, and S_ACN_ were previously reported, but a description of some key topographical features is provided here for discussion purposes. In all three forms, NCL molecules assemble into π-stacks along the *a*-axis. Molecules within a π-stack are related by translation with repeat distances of 3.81, 3.74, and 3.83 Å in H_A_, S_MeOH_, and S_ACN_, respectively. The edges of the π-stacks define the perimeter of one-dimensional solvent channels. Packing diagrams of each structure are shown in Figure 2. The left view is down the π-stacking and channel axis (*a*-axis). The middle view is down the *b*-axis, with the channel in the horizontal direction. The right view is a close-up showing the NCL···solvent hydrogen-bonding interactions. Importantly, though H_A_, S_MeOH_, and S_ACN_ are all one-dimensional channel solvates, none are isostructural.

H_A_ and S_MeOH_ share some similar topological features. When viewed down the channel axis (*a*-axis), each water or methanol is surrounded by three neighboring NCL stacks with the same relative orientation. Solvent molecules act as a hydrogen-bond acceptor to a phenol O_(H_2_O/MeOH)_···H–O and a hydrogen-bond donor to a nitro H_(H_2_O/MeOH)_···O_2_N group. Water has a third hydrogen bond to H_(H_2_O)_···O=C, which is not possible in S_MeOH_. Hydrogen-bond distances are similar (see Table 1). The structural differences in the two forms become more apparent when they are viewed down the *b*-axis. In H_A_, all π-stacks tilt in the same direction with respect to the (001) plane, whereas in S_MeOH_, the tilt direction alternates in adjacent (001) layers owing to a 2_1_ screw along the *c*-axis.

**Table 1:** Structural Data for H_A_, S_MeOH_, and S_ACN_ and Solvent-Free F1

	H_A_	S_MeOH_	S_ACN_	F1
refcode	OBEQAN01	GOJLIC	KUKROZ	HEBFUR
*T* (K)	RT	100	120	100
solvent	H_2_O	CH_3_OH	C_2_H_3_N	
space group	*P*2_1_/*c*	*P*2_1_2_1_2_1_	P-1	*P*2_1_/*c*
*a* (Å)	3.813	3.740	3.830	7.067
*b* (Å)	16.143	17.420	11.732	13.485
*c* (Å)	23.065	22.180	16.903	13.510
α (deg)	90	90	87.98	98.34
β (deg)	92.87	90	87.12	90
γ (deg)	90	90	84.08	90
vol (Å^3^)	1418.08	1445.05	754.20	1273.83
*Z*	4	4	2	4
solvent···NCL	O···H–O (2.66 Å); O–H···O_2_N (3.00 Å); O–H···O=C(2.74 Å)	O···H–O (2.52 Å); O–H···O_2_N (3.28 Å)	CN···H–O (2.78 Å)	
NCL π-stack	3.81 Å^a^	3.74 Å^a^	3.83 Å^a^	3.53 Å^b^
solvent bp	100 °C	64.7 °C	81.6 °C	
solvent fraction of unit cell volume^c^	7.3%	12.0%	17.8%	

a Related by translation.

b Related by 2-fold rotation.

c Calculated using Solvate Analyzer with a 1.0 Å probe radius.

In S_ACN_, the one-dimensional solvent channels differ in that the perimeter is defined by the edges of four NCL π-stacks. Acetonitrile molecules interact with the phenols of one stack via an N_(ACN)_···H–O hydrogen bond; however, the other three NCL π-stacks that define the channel have a different relative orientation than those in H_A_ and S_MeOH_. Notably, a larger fraction of the S_ACN_ channel surface exposes chlorine atoms, presumably as a means to locally compensate for the larger dipole moment of the included acetonitrile molecules. The view along the *b*-axis shows that all NCL π-stacks tilt in the same direction, though the layers are interdigitated with chlorophenol rings and nitroaromatic rings segregated into different (00*l*) planes.

### Solid-State Desolvation

With three unique channel solvate topologies, a comparative analysis of the desolvation mechanisms under similar conditions (0% RH, heat) affords the opportunity to assess potential correlations between the lattice and the transformation mechanism. Desolvation entails both solvent loss and (typically) some lattice rearrangement to a solvent-free form. But open questions remain, including whether solvent loss occurs prior to or simultaneously with the lattice change and whether intermediate crystalline forms are involved. The solvent boiling points (methanol < acetonitrile < water) and volume fraction of the unit cell (water < methanol < acetonitrile) follow different trends. Solvent volumes estimated using the Solvate Analyzer^40,41^ tool in Mercury and a 1.0 Å probe radius were 7.3% in H_A_, 12.0% in S_MeOH_, and 17.8% in S_ACN_ (Figure S2).

In previous work, we investigated^28^ the molecular-level dehydration process of H_A_ using complementary thermogravimetric kinetic studies to track the rate of solvent loss and time-resolved synchrotron powder X-ray diffraction to elucidate structural changes in real time. When heated under 0% RH, H_A_ was found to lose water via diffusion to create an isomorphous anhydrate (H*). Though water loss ruptures the hydrogen-bonding interactions and leaves NCL hydrogen-bond donor and acceptor groups unsatisfied, H* was stable to well above the dehydration temperature. Only at temperatures >150 °C did H* undergo a spontaneous and rapid transformation to the higher-density F1 polymorph. A least-motion pathway from H* to F1 was proposed based on the highly anisotropic thermal expansion of H* and the topology and symmetry within the π-stacks of the two forms.

With larger solvent channel dimensions, different layer orientations, and/or different lattice topologies, elucidating desolvation processes in S_MeOH_ and S_ACN_ under similar environmental conditions affords some interesting comparisons. S_MeOH_ and S_ACN_ are discussed in separate sections below.

### Desolvation of S_MeOH_

**Figure 3:**
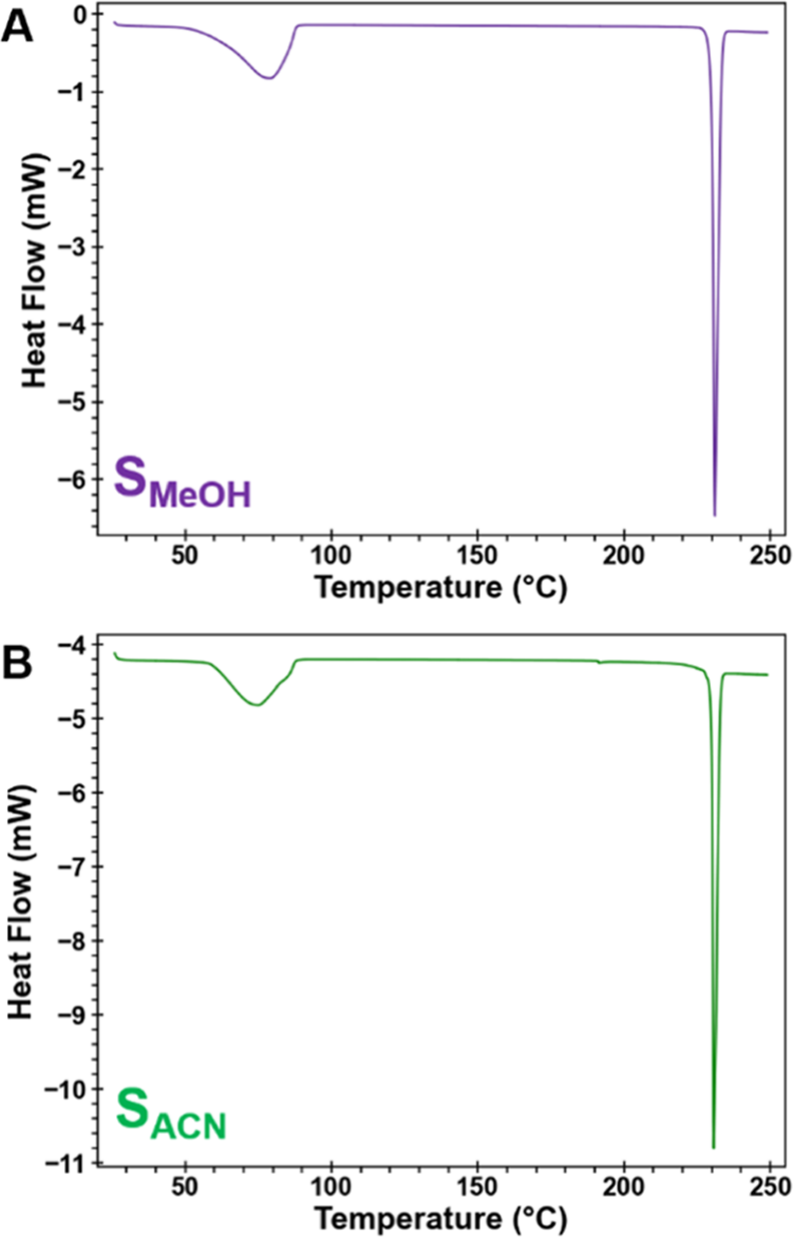
DSC thermograms of hand-ground S_MeOH_ (A) and (B) S_ACN_.

The DSC thermogram of S_MeOH_ has two endothermic transitions (Figure 3A). The first, with a *T*_max_ = 77.4 ± 1.1 °C can be attributed to desolvation, and the higher one at 230.8 ± 0.2 °C to the melting of F1. There was no statistical difference in the temperatures of ground and unground samples (Figure S3). Under TGA heating conditions, S_MeOH_ weight loss was 7.7 ± 0.1% (calc. 8.9%) with the greatest weight loss occurring between 58 and 75 °C (Figure S4). This means 1.2 wt % loss (∼13.5% of the methanol in the monosolvate) occurs in the time between harvesting the crystals from the growth solution and the start of the TGA. Our S_MeOH_ desolvation temperatures are in reasonable agreement with previously reported values by Van Tonder et al.^29^ who cited DSC desolvation (pinhole pans) at 90 ± 3 °C but also noted that crystals begin to darken at ∼60 °C under hot stage microscopy conditions.

**Figure 4:**
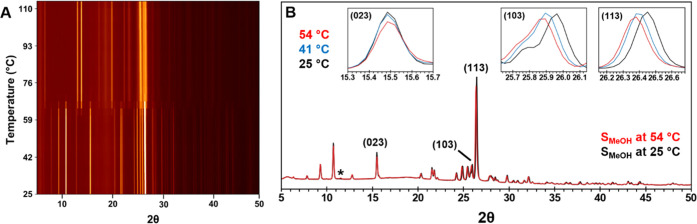
(A) Contour plot of S_MeOH_ heated at 10 °C/min from room temperature to 115 °C. The 2θ scale is normalized to Cu Kα. (B) Superposition of sPXRD patterns collected at 25 and 54 °C. The peak labeled (*) does not correspond to either S_MeOH_ or F1 and only appears between 44 and 65 °C. Inset plots are close-ups of individual peaks in diffraction patterns collected at 25, 41, and 54 °C.

The desolvation of S_MeOH_ was investigated with time-resolved in situ sPXRD by heating at 10 °C/min to 115 °C and isothermally at 40, 45, and 50 °C under a constant He flow (RH = 0%). Continuous data acquisition enabled high-resolution patterns to be obtained every ∼20 s. A representative contour plot of S_MeOH_ desolvation under temperature ramping conditions is shown in Figure 4A. Comparison of the sPXRD pattern against the pattern simulated from the single-crystal structure confirmed the sample phase purity at the start of the experiment (Figure S5). This was important to verify in light of a previous work by Harriss et al.^24^ who observed changes in the PXRD pattern of S_MeOH_ stored in air overnight. They ascribed the changes to the partial conversion of the sample to H_A_.

As S_MeOH_ is heated, subtle changes are observed in the positions and intensities of some sPXRD lines up to ∼58 °C (Figure 4B). Peak position shifts to lower 2θ values can be explained by thermal expansion. Small intensity changes are likely due to partial solvent loss, although peak intensities can also change in response to temperature differences. For example, between 25 and 54 °C, the (023), (103), and (113) reflections decreased by 12.0, 5.5, and 8.1%, respectively (Figure S6). Decreased intensities of the (023) and (103) peaks were predicted by comparing the simulated PXRD patterns of S_MeOH_ (GOJLIC) and a modified CIF file with the methanol removed. At ∼44 °C, a new low-intensity peak also appears at 2θ = 11.524° (labeled *), which does not correspond to F1 or H_A_/H*. However, this peak was short-lived and was not reproduced in isothermal experiments. At ∼58 °C, multiple peaks corresponding to F1 become evident, which is consistent with the temperature at which rapid weight loss is observed in TGA. As heating continues, the intensities of the F1 peaks increase and the S_MeOH_ peaks decrease until by 71 °C only F1 remains (Figure S7). From the time F1 first appears, the S_MeOH_ to F1 transformation reaches completion within ∼2 min.

Isothermal desolvation at 40, 45, and 50 °C proceeded similarly (Figure S8), though the time needed for complete transformation to F1 was longer. The reaction times at 50 and 45 °C were ∼13 and 20 min, respectively. At 40 °C, the time between the first appearance of F1 and the complete disappearance of S_MeOH_ was ∼51 min.

**Figure 5:**
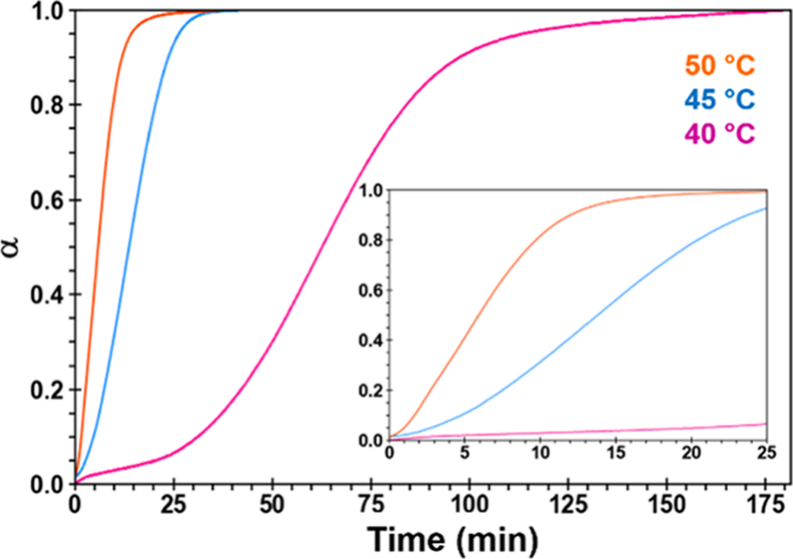
Fraction of the total measured TGA weight loss (α) vs time plot for S_MeOH_ based on triplicate TGA data collected at 40, 45, and 50 °C. The inset plot is an expanded view of the data from 0–25 min.

Complementary TGA kinetics experiments on S_MeOH_ were performed at 40, 45, and 50 °C, yielding the representative plot of the fraction of the measured TGA weight loss (α) vs time shown in Figure 5. Solvent loss prior to the start of the TGA experiment, including during sample prep and the time needed to reach each holding temperature, is not included in the plot. The reaction conversion data in the linear regions (0.1 < α < 0.9) were fit to 17 different solid-state reaction models^31^ (Table S2). The mathematical models can aid in identifying the rate-limiting step of the reaction, which could be the conversion of the reacting solvate (geometric contraction), growth of the solvent-free product (nucleation), or rate of solvent loss (diffusion). TGA data were a good fit (*R*^2^ > 0.99) to several nucleation and geometric contraction models, though the data proved to be a poor fit to all diffusion models.

The measured rate constant for the desolvation reaction at the three isothermal temperatures was 0.021, 0.064, and 0.12 min^–1^. When fit to an Arrhenius plot, this yielded *E*_a_ = 145 ± 26 kJ/mol. Model-free methods were also used to assess whether the activation energy changes over the course of the reaction (Figure S9). This entails plotting ln(dα/d*t*)_α_ as a function of 1/*T* (Friedman method) or plotting −ln(*t*)_α_ as a function of 1/*T* (standard method). Both methods indicated the activation energy decreases as a function of the reaction progress. This is consistent with a transformation that is rate-limited by the surface area of the reaction front, which decreases with time.

### Desolvation of S_ACN_

To our knowledge, the desolvation behavior of S_ACN_ has not previously been reported, though Sovago and Bond^21^ note that when S_ACN_ was removed from the growth solution and the solvent was allowed to evaporate completely, “H_A_ was frequently observed to form.” Our DSC thermograms (unsealed pans) of S_ACN_ showed two endothermic transitions (Figure 3B). The first, with a *T*_max_ = 76.6 ± 1.3 °C can be attributed to desolvation, and the higher one at 230.6 ± 0.1 °C to melting of the F1 anhydrate. Unground and manually ground samples heated under the same conditions had comparable dehydration and melting temperatures. Under TGA heating conditions, weight loss of 9.5 ± 0.1% (calc. 11.1%) was observed between 55 and 74 °C (Figure S4). The difference in the measured and calculated weight loss is notable, with the premature loss of 1.6 wt % (∼14.4% of the acetonitrile in the monosolvate) occurring in the time between harvesting the crystals from the growth solution and the start of the TGA analysis.

**Figure 6:**
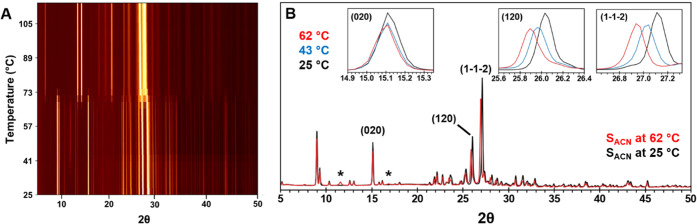
(A) Contour plot of ground S_ACN_ heated at 10 °C/min from room temperature to 115 °C. The 2θ scale is normalized to Cu Kα. (B) Superposition of the sPXRD patterns at 25 and 62 °C. The peaks labeled (*) do not correspond to either S_ACN_ or F1 and exist transiently between 32 and 76 °C. Inset plots correspond to (020), (120), and (1–1–2) peaks in diffraction patterns collected at 25, 43, and 62 °C.

In situ synchrotron PXRD was used to track the structural changes that occur upon desolvation. Comparison of the sPXRD pattern against the pattern simulated from the single-crystal structure confirmed the sample phase purity at the start of each experiment (Figure S10), which suggests that early solvent loss likely occurs via diffusion. A representative contour plot of S_ACN_ when heated at 10 °C/min is shown in Figure 6A. As in S_MeOH_, some S_ACN_ reflections undergo slight changes in position and intensity due to thermal expansion and presumably additional acetonitrile loss. The sPXRD patterns of S_ACN_ at 25 and 62 °C are superimposed in Figure 6B with (020), (120), and (103) diffraction lines indicated. In this temperature range, these lines decreased by 18.7, 24.5, and 18.6%, respectively. This is consistent with changes anticipated by comparing the simulated pattern from the cif and a modified cif with acetonitrile removed. At ∼33 °C in one experiment, two unassignable low-intensity peaks at 2θ = 11.524° and 16.805° were observed transiently. All other peaks in sPXRD patterns correspond to the S_ACN_ lattice up to 65 °C, the temperature at which F1 is first observed. This is close to the temperature where rapid weight loss occurs in TGA. With further heating, the F1 peaks grow in intensity until it is the only phase remaining at 76 °C. The S_ACN_-to-F1 transformation proceeded more slowly when isothermally heated at 40 and 45 °C. At these temperatures, once F1 first appeared the phase change reached completion after ∼31 and 20 min, respectively (Figure S12).

**Figure 7:**
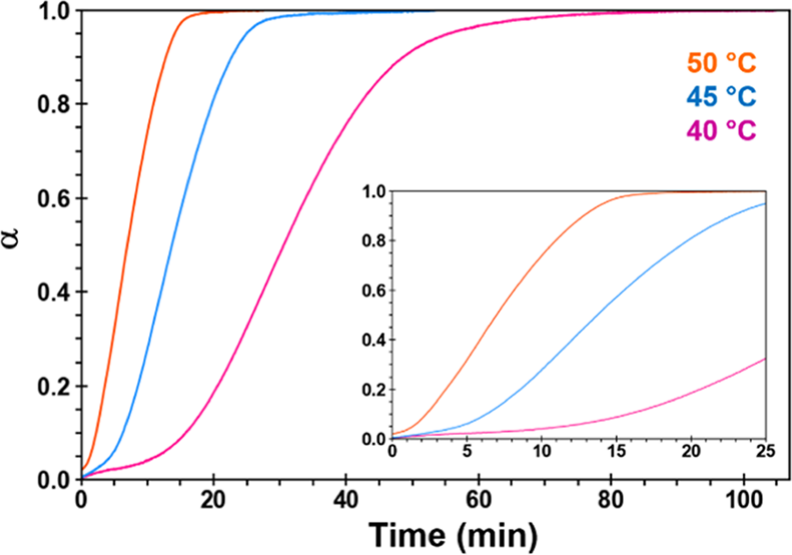
Fraction of the measured TGA weight loss (α) vs time plot for S_ACN_ based on triplicate TGA data collected at 40, 45, and 50 °C. The inset plot is an expanded view of the data from 0–25 min.

A plot of the fraction of the measured TGA weight loss (α) vs time based on complementary TGA kinetics data at 40, 45, and 50 °C appears in Figure 7. Solvent loss prior to reaching the holding temperature is not included in the plot. The linear regions (0.1 < α < 0.9) were fit to different solid-state reaction models, and the top model at all three temperatures was one-dimensional nucleation (A2) with an *R*^2^ > 0.999 (Table S3). With rate constants of 0.035, 0.077, and 0.11 min^–1^ at these three temperatures, an Arrhenius plot yielded an *E*_a_ = 94.7 ± 26 kJ/mol (Figure S13).

#### Comparison of H_A_, S_MeOH_, and S_ACN_ Desolvation

In each of the three solvates shown in Figure 2, solvent molecules occupy one-dimensional channels created by the void space between face–face π-stacked NCL molecules. Desolvation in each system yields the same F1 product, although experiments performed under similar conditions indicate that the rate-limiting step is not the same. This provides a unique opportunity to consider which physical or structural aspects have the strongest influence on the solid-state desolvation process.

Evidence indicates some solvent loss from each of the three forms prior to the formation of F1. For H_A_, water loss is complete by ∼85 °C (in TGA), but the hydrate lattice persists to 140 °C or higher in sPXRD (Figure S14). This was strong evidence for the formation of an anhydrous isomorphous intermediate. In S_MeOH_ and S_ACN_, the desolvation temperature (by TGA) and the temperature at which F1 appeared (by sPXRD) are in much closer agreement. TGA also consistently showed a lower actual wt % loss than the theoretical wt % in S_MeOH_ and S_ACN_. Yet at the start of sPXRD experiments, each solvate appeared to be phase-pure. This coupled with additional decreases in the relative intensity of select diffraction lines in S_MeOH_ and S_ACN_ suggest each solvate lattice has a substoichiometric solvent content at the time F1 first appears. This early solvent loss must occur via diffusion, as other mechanisms would be expected to result in the appearance of new diffraction lines.

Water (100 °C), methanol (64.7 °C), and acetonitrile (81.6 °C) have very different boiling points, though the onset temperature for rapid weight loss begins at very similar temperatures (*T*_onset_ = 58 °C for S_MeOH_ and *T*_onset_ = 55 °C in H_A_ and S_ACN_). Therefore, T_onset_ is not strongly correlated with the solvent’s boiling point or the number of solvent···NCL hydrogen-bonding interactions. Differences in the hydrogen-bonding environment and volatility of each solvent likely do play a role in the reaction kinetics, as complete desolvation requires qualitatively longer times in H_A_ (complete by 85 °C) than in S_MeOH_ and S_ACN_ (complete by 74–75 °C). This is also reflected in the rate constants derived from isothermal experiments at 40, 45, and 50 °C, where H_A_ < S_MeOH_ ≈ S_ACN_.

We speculate that one reason S_MeOH_ and S_ACN_ do not yield solvent-free isomorphs is that these hypothetical forms may not be sufficiently dense to exist as stable intermediates. To estimate the magnitude of the density change on going from a solvate to a fully desolvated isomorph, the packing fraction (PF) was calculated for each CIF file listed in Table 1 and modified CIFs with the solvent removed. All three solvates have PFs in the typical range for molecular crystals,^42^ with H_A_ = 71.8%, S_MeOH_ = 73.6%, and S_ACN_ = 74.0%. Hypothetical isomorphs with the solvent completely removed would have PFs of 67.9% (H*), 65.3%, and 62.8%, respectively. If just half of the methanol and acetonitrile molecules were removed, the PF of hypothetical hemisolvates would be higher than the PF of H*, although it seems unlikely that even a hemisolvate isomorph can be reached. While early methanol and acetonitrile loss can occur without significant lattice changes, this appears to be feasible only to a point.

**Figure 8:**
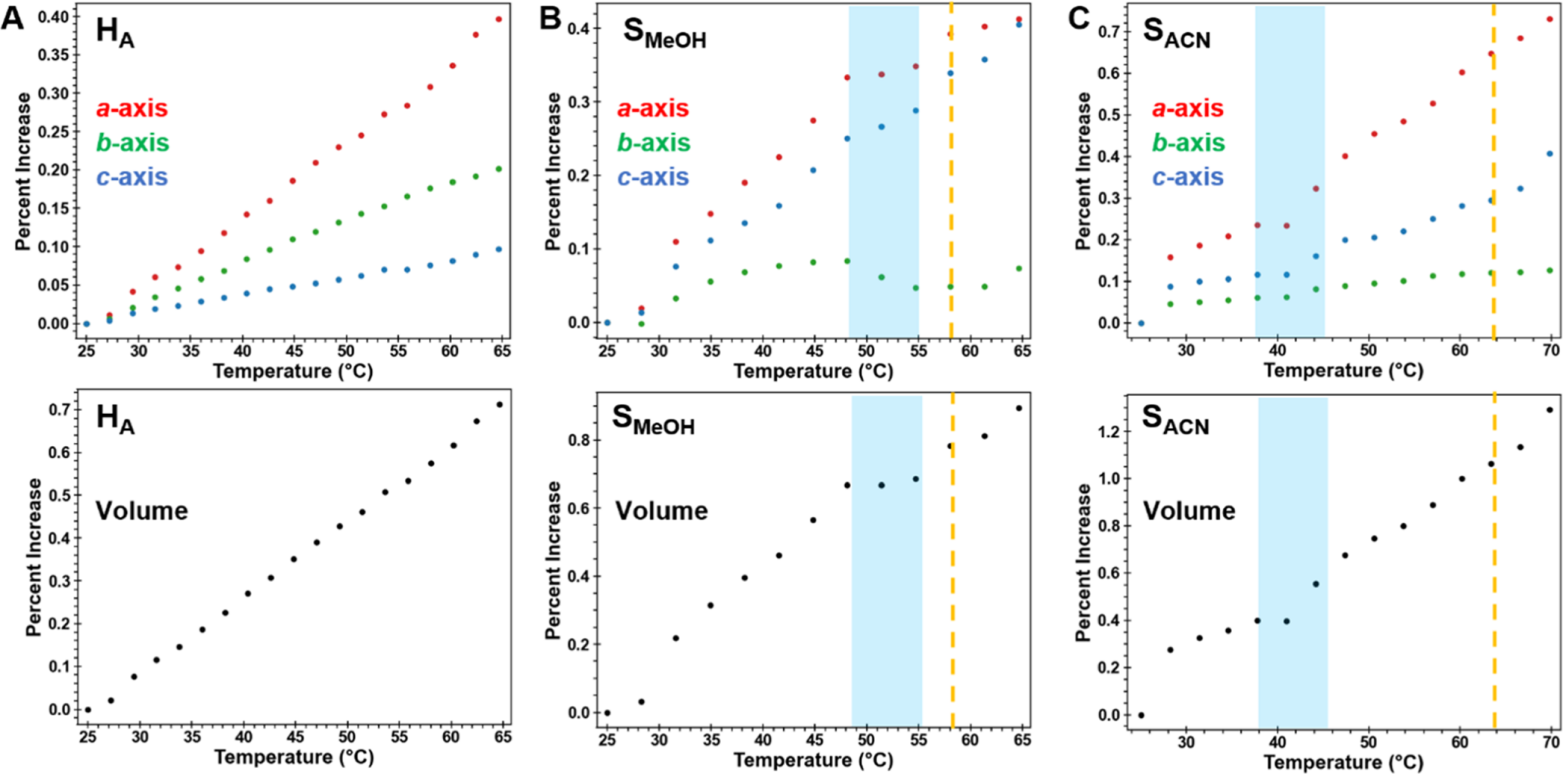
Thermal expansion in (A) H_A_, (B) S_MeOH_, and (C) S_ACN_ between 25 and 65 °C determined from sequential Pawley refinement of sPXRD data. The shaded blue region is where a change in relative expansion of the three axes occurs. The dashed orange lines mark the temperature where F1 first appears. The 2θ range is shown on a Cu Kα scale.

Yet density factors alone are an incomplete and too-simplified rationale for the different desolvation mechanisms adopted by S_MeOH_ and S_ACN_ compared to H_A_. Comparison of the thermal expansion in H_A_, S_MeOH_, and S_ACN_ reveals some additional complexities associated with the desolvation process. The refined cell axes and volumes of S_MeOH_ and S_ACN_ lattices between 25 and 65 °C are shown in Figure 8, where they are compared to H_A_ over the same temperature range. The dashed orange line indicates the temperature at which F1 is first observed. Notably, in S_MeOH_ and S_ACN_, the cell volume expands at a much faster rate than in H_A_ (e.g., between 25 and 50 °C, S_MeOH_ and S_ACN_ increase by >0.65% but H_A_ by only 0.44%).

As previously reported, in H_A_ water loss yields an isomorphous desolvate, H*, which is stable to at least 140 °C.^28^ The rate of thermal expansion along the three axes in H_A_ (below 55 °C) and H* (above 85 °C) is continuous over the entire heating range (Figure S15). A smooth transition from H_A_ to H* to F1 was facilitated by anisotropic thermal expansion, which was largest along the π-stacking direction. As the repeat distance between molecules in the π-stacks increases, eventually the cooperative rotation of molecules forms the one-dimensional hydrogen-bonded chains in F1. In contrast, some sudden changes in the S_MeOH_ and S_ACN_ lattices upon heating are apparent, even prior to the appearance of the F1 product.

As in H_A_, the largest thermal expansion in S_MeOH_ and S_ACN_ is also along the π-stacking direction (*a*-axis). However, expansion along *c* > *b* in S_MeOH_ and S_ACN_ occurs, while expansion along *b* > *c* in H_A_. In S_MeOH_, the three cell axes steadily increase between 30 and 48 °C, but the *a-*axis plateaus and the *b*-axis actually shortens between 48 and 55 °C. The plateau is also apparent in the cell volume. Soon after, the diffraction lines corresponding to F1 first appear. This plateauing suggests a critical point is reached, where continued expansion at the same rates along the three axes is no longer the most energetically feasible path. We speculate this could be related to either reaching a critical level of solvent loss or other structural factors such as “pinning” at the interface between the dense (001) layers.

A similar, albeit more subtle, shift is seen in S_ACN_. Cell parameters steadily increase at the lower temperatures but plateau briefly between 38 and 42 °C. The α, β, and γ angles also abruptly change in this temperature region (Figure S16). Once the lattice adjusts, above 42 °C, the rate of thermal expansion in the *a*- and *c*-axis directions is much higher than it was below 38 °C. The α, β, and γ angles also continuously change until F1 first appears at ∼65 °C. As in S_MeOH_, we interpret these sudden lattice changes to be an indication that through some combination of thermal expansion, solvent loss, or pinning effects, a critical point is reached, which alters the trajectory of the desolvation process. The structural changes in S_ACN_ begin at a temperature well below that when F1 first appears, which we speculate may be related to greater interdigitation of the (001) layers.

## Conclusions

Just as water always rolls downhill, solid-state transformations are expected to follow the lowest energy path available. Yet it is exceedingly difficult to predict the low energy trajectory of a solid-state desolvation reaction when both the chemical composition and the lattice parameters change over the reaction coordinate. Insight into the molecular-level mechanisms of such processes can be gained by employing complementary time-resolved methods that can probe both the solvent loss and how the lattice structure responds. Comparisons across channel solvates S_MeOH_, S_ACN_, and H_A_ are informative since each reaction starts with a different NCL topology but ends with the same final solvent-free form. As this work illustrates, the molecular-level route taken by each of these solvates differs in interesting and sometimes surprising ways.

With each water molecule in H_A_ triply hydrogen-bonded to NCLs, it was not obvious that water diffusion along a one-dimensional channel was even possible, or that an isomorphous dehydrated intermediate would persist to much higher temperatures. Our initial predictions that similar behavior would be observed in S_MeOH_ and S_ACN_ channel solvates were only partially correct. In both S_MeOH_ and S_ACN_, some solvent loss initially occurs presumably by diffusion, but this appears to be viable for only a fraction of solvent molecules. The inability of S_MeOH_ and S_ACN_ to form an isomorphous solvent-free intermediate is likely due to a combination of factors, including but not limited to density and pinning of layers, which induce other structure changes. It is difficult to make predictions of what might happen if desolvation were performed under different operating conditions (e.g., relative humidity, heating rate, particle size), though exploration of process controls may yield some additional insights.

## Supporting Information Available

Micrographs, kinetics tables, thermal plots (DSC and TGA), and synchrotron PXRD data (PDF)
