# Supplementary material for: Desolvation Processes in Channel Solvates of Niclosamide
Source: Mol Pharm. 2023 Oct 18;20(11):5554–62. doi: 10.1021/acs.molpharmaceut.3c00481 (PMC10630950; doi:10.1021/acs.molpharmaceut.3c00481)
Supplement: Supplementary file 1 — mp3c00481_si_001.pdf [file mp3c00481_si_001.pdf]

Supporting Information

Desolvation Processes in Channel Solvates of Niclosamide

Jen E. Mann, Renee Gao, Shae S. London and Jennifer A. Swift*

Georgetown University, Department of Chemistry, Washington, DC 20057-1227

Table of Contents **Page**

Figure S1. Optical micrographs of S_{MeOH}, S_{MeOH} and H_A. Each form crystalizes as needles but with slightly different aspect ratios. 3

Table S1. Solid state reaction models and integral expressions used for kinetic analyses. 3

Figure S2. Solvent channels in H_A, S_{MeOH}, and S_{ACN} viewed with the Mercury Solvate Analyzer tool using a probe radius of 1.0 Å and a grid spacing of 0.3 Å. Each solvate is viewed along and normal to the channel axis. 4

Figure S3. DSC curves of heat flow vs. temperature for H_A, S_{MeOH} and S_{ACN} that were (A, B, C) hand-ground with a mortar and pestle or (D, E, F) unground. All samples were heated at 5 °C/min to 250 °C in aluminum pans with unsealed lids. 4

Figure S4. TGA of heat flow vs. temperature for (A) S_{MeOH} and (B) S_{ACN}. Samples were heated at 5 °C/min in open pans. 5

Figure S5. Experimental sPXRD of S_{MeOH} at the start of the dehydration experiment compared against the simulated PXRD from the single crystal structure. 5

Figure S6. Change in peak intensity for (023), (103), and (113) diffraction lines of S_{MeOH} with temperature. From 25 to 54 °C, the peaks decrease by 12.0%, 5.5%, and 8.1%, respectively. 6

Figure S7. Experimental sPXRD of the S_{MeOH} dehydration product at 71 °C and 115 °C compared against the simulated PXRD of the F1 single crystal structure. 6

Figure S8. Contour plots of S_{MeOH} heated to and held isothermal at (A) 40, (B) 45, and (C) 50 °C. Samples were either loaded into the capillary as a wet paste (40 and 45 °C) or loaded wet (50 °C). S_{MeOH} was able to completely transition to phase pure Form 1 after approximately 51, 20, and 13 minutes at 40, 45, and 50 °C. 7

Table S2. Correlation coefficients associated with different solid state reaction models for $\mathbf{S_{MeOH}}$ (ground) isothermal TGA data (40, 45, and 50 °C). Reaction models with $R^2 > 0.99$ are red and with $R^2 \geq 0.999$ are red and bold. 8

Figure S9. E_a values determined from model-based and model-free kinetic analyses of S_{MeOH} TGA isothermal desolvation at 40, 45 and 50°C. (A) The three nucleation models with the highest R^2 values (A2, A3 and B1) yielded similar E_a values. (B) Representative Arrhenius plot of the A2 model. Time-dependent E_a values calculated from model-free (C) Friedman and (D) Standard methods show a decreasing E_a as a function of reaction progress. **9**

Figure S10. **E**xperimental sPXRD of S_{ACN} at the start of the dehydration experiment compared against the simulated PXRD from the single crystal structure. **10**

Figure S11. Change in peak intensity for (020), (120), and (1-1-2) diffraction lines of S_{ACN} with temperature. From 25 to 62 °C, the peaks decrease by 18.7%, 24.5%, and 18.6%, respectively. **10**

Figure S12. Contour plots of S_{ACN} heated to and held isothermal at (A) 40 ℃ and (B) 45 ℃. Samples were loaded into the capillary as a wet paste (40 and 45 ℃) and were able to completely transition to phase pure Form 1 after approximately 31 and 20 minutes, respectively. **11**

Table S3. Correlation coefficients associated with different solid state reaction models for $\mathbf{S_{ACN}}$ (ground) isothermal TGA data (40, 45, and 50 °C). Reaction models with $R^2 > 0.99$ are red and with $R^2 \geq 0.999$ are red and bold. **12**

Figure S13. E_a values determined from model-based and model-free kinetic analyses of S_{ACN} TGA isothermal desolvation at 40, 45 and 50°C. (A) The three nucleation models with the highest R^2 values (A2, A3 and B1) yielded similar E_a values. (B) Representative Arrhenius plot of the A2 model. Time-dependent E_a values calculated from model-free (C) Friedman and (D) standard analysis methods. **13**

Figure S14. Comparison of TGA of heat flow vs. temperature for (A) S_{MeOH}, (B) S_{ACN} and (C) H_A collected under identical conditions (heating rate = 5 ℃/min, open pans). **13**

Figure S15. Thermal expansion in H_A and H* over the temperature range up to 150 °C. **14**

Figure S16. Pawley refined cell parameters for S_{ACN} based on sPXRD desolvation data. **14**

Figure S17. ^{1}H NMR ($CDCl_3$) of dissolved S_{MeOH} and S_{ACN} crystals. **15**

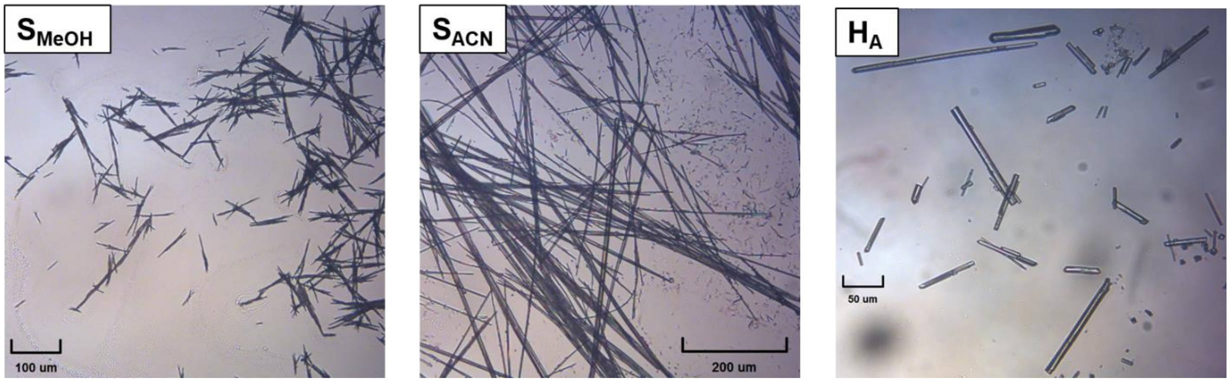

Figure S1. Optical micrographs of S_{MeOH}, S_{MeOH} and H_A. Each form crystalizes as needles but with slightly different aspect ratios.

Table S1. Solid state reaction models and integral expressions used for kinetic analyses.

**Desolvation Models**	**Integral Equation** $g(\alpha) = kt$
**Nucleation Models**	
1D growth of nuclei – Avrami-Erofeyev(**A2**)	$(-\ln(1-\alpha))^{0.5}$
2D growth of nuclei – Avrami-Erofeyev (**A3**)	$(-\ln(1-\alpha))^{1/3}$
3D growth of nuclei – Avrami-Erofeyev (**A4**)	$(-\ln(1-\alpha))^{1/4}$
Random nucleation – Prout-Tompkins (**B1**)	$\ln(\alpha/(1-\alpha)) + e^{\alpha}$
Power law (**P2**)	$\alpha^{1/2}$
Power law (**P3**)	$\alpha^{1/3}$
Power law (**P4**)	$\alpha^{1/4}$
**Geometrical Contraction Models**	
2D phase boundary / contracting area (**R2**)	$1-(1-\alpha)^{1/2}$
3D phase boundary / contracting volume (**R3**)	$1-(1-\alpha)^{1/3}$
**Diffusion Models**	
1D diffusion (**D1**)	α^2
2D diffusion (**D2**)	$(1-\alpha)*(\ln(1-\alpha))+\alpha$
3D diffusion - Jander (**D3**)	$(1-(1-\alpha)^{1/3})^2$
3D diffusion – Ginstling - Brounshtein (**D4**)	$(1-(2/3)*\alpha)-(1-\alpha)^{2/3}$
**Reaction Order Models**	
Zero-order (**R1**)	α
First-order (**F1**)	$-\ln(1-\alpha)$
Second-order (**F2**)	$(1/(1-\alpha))-1$
Third-order (**F3**)	$(1/2)*(((1-\alpha)^{-2})-1)$

Adapted from: Khawam, A.; Flanagan, D. R., Solid-State Kinetic Models: Basics and Mathematical Fundamentals. *J. Phys. Chem. B* **2006,** *110* (35), 17315-17328.

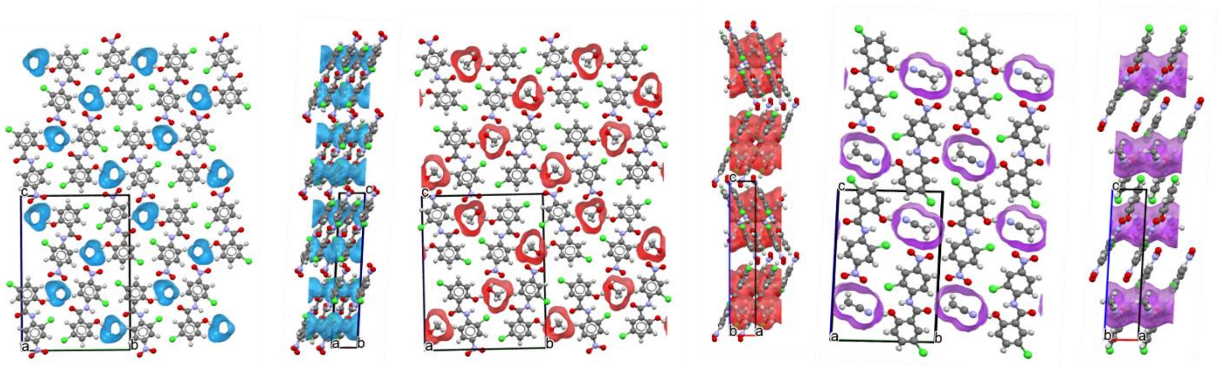

Figure S2. Solvent channels in (left) H_A, (middle) S_{MeOH}, and (right) S_{ACN} viewed with the Mercury Solvate Analyzer tool using a probe radius of 1.0 Å and a grid spacing of 0.3 Å. Each solvate is viewed along and normal to the channel axis.

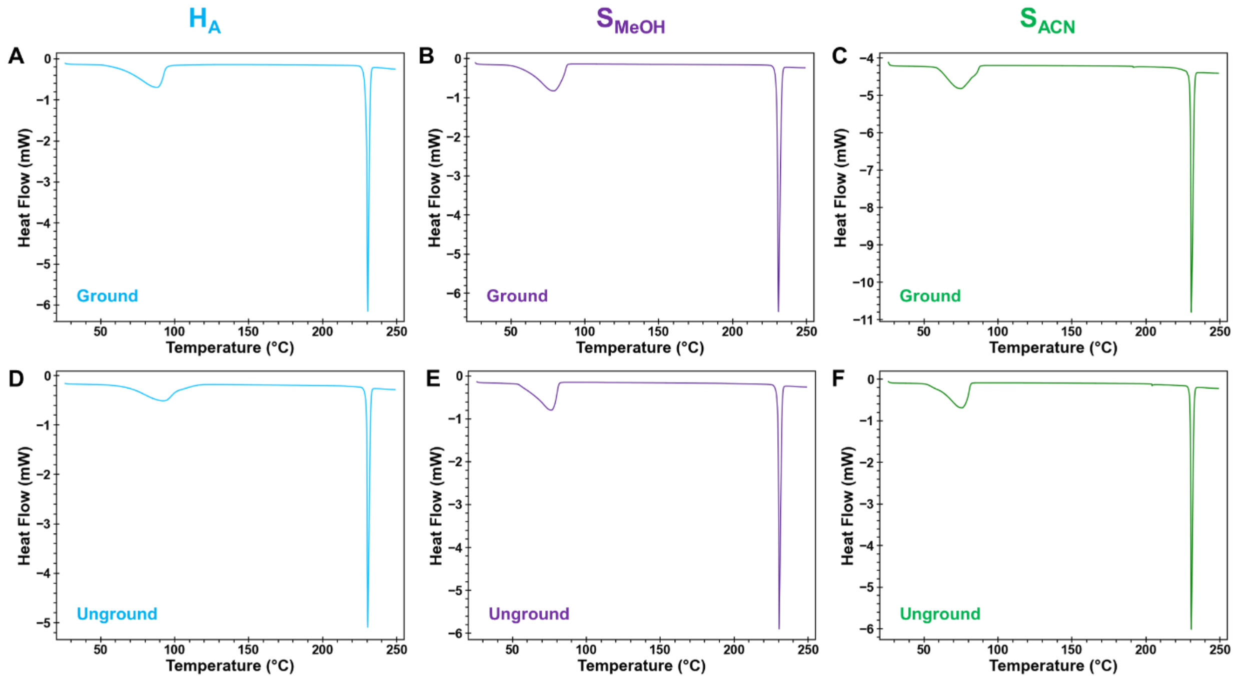

Figure S3. DSC curves of heat flow vs. temperature for H_A, S_{MeOH} and S_{ACN} that were (A, B, C) hand-ground with a mortar and pestle or (D, E, F) unground. All samples were heated at 5 °C/min to 250 °C in aluminum pans with unsealed lids.

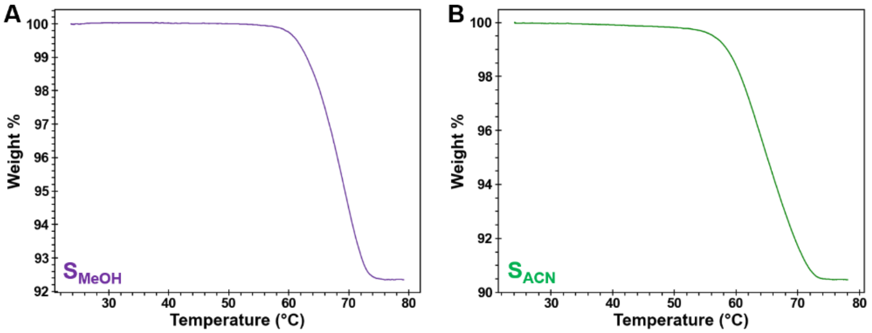

Figure S4. TGA of heat flow vs. temperature for (A) S_{MeOH} and (B) S_{ACN}. Samples were heated at 5 °C/min in open pans.

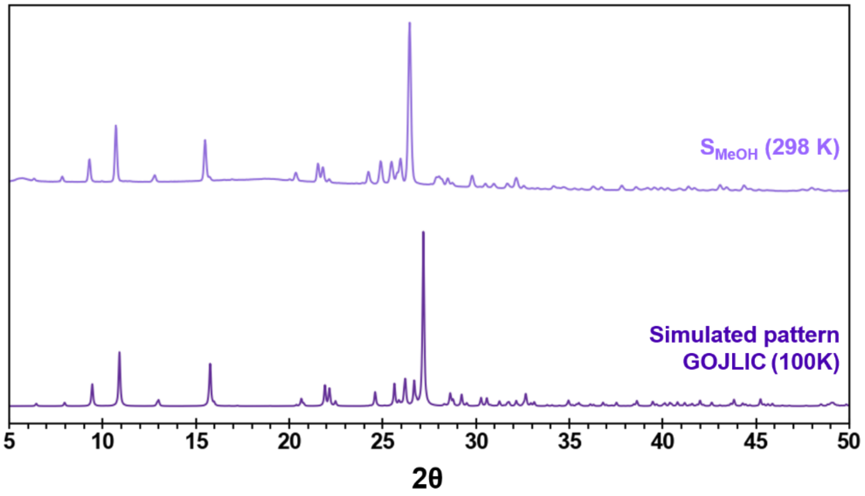

Figure S5. Experimental sPXRD of S_{MeOH} at the start of the dehydration experiment compared against the simulated PXRD from the single crystal structure.

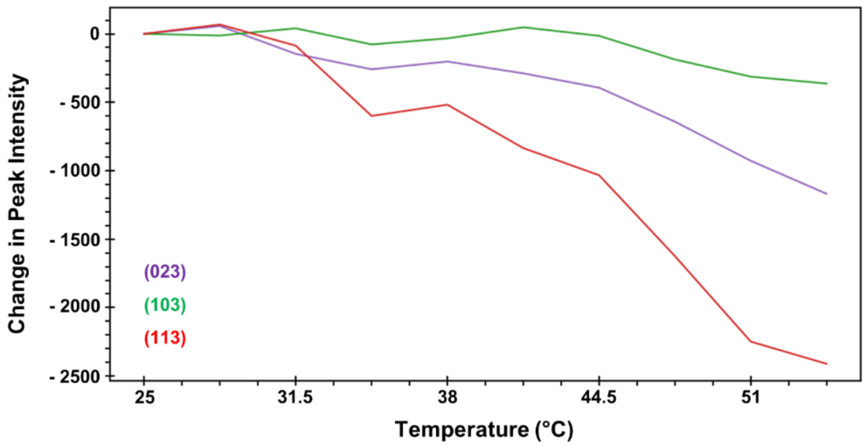

Figure S6. Change in peak intensity for (023), (103), and (113) diffraction lines of S_{MeOH} with temperature. From 25 to 54 °C, the peaks decrease by 12.0%, 5.5%, and 8.1%, respectively.

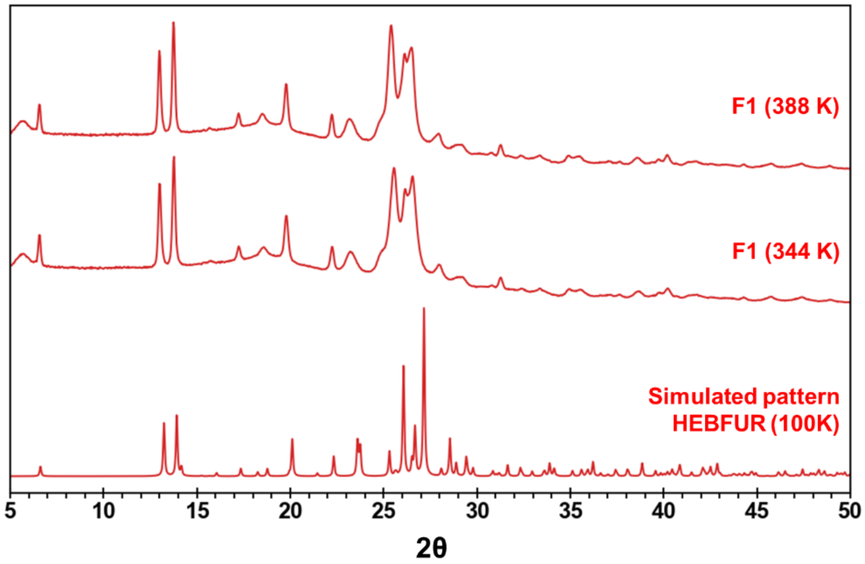

Figure S7. Experimental sPXRD of the S_{MeOH} dehydration product at 71 °C (344 K) and 115 °C (388K) compared against the simulated PXRD of the F1 single crystal structure.

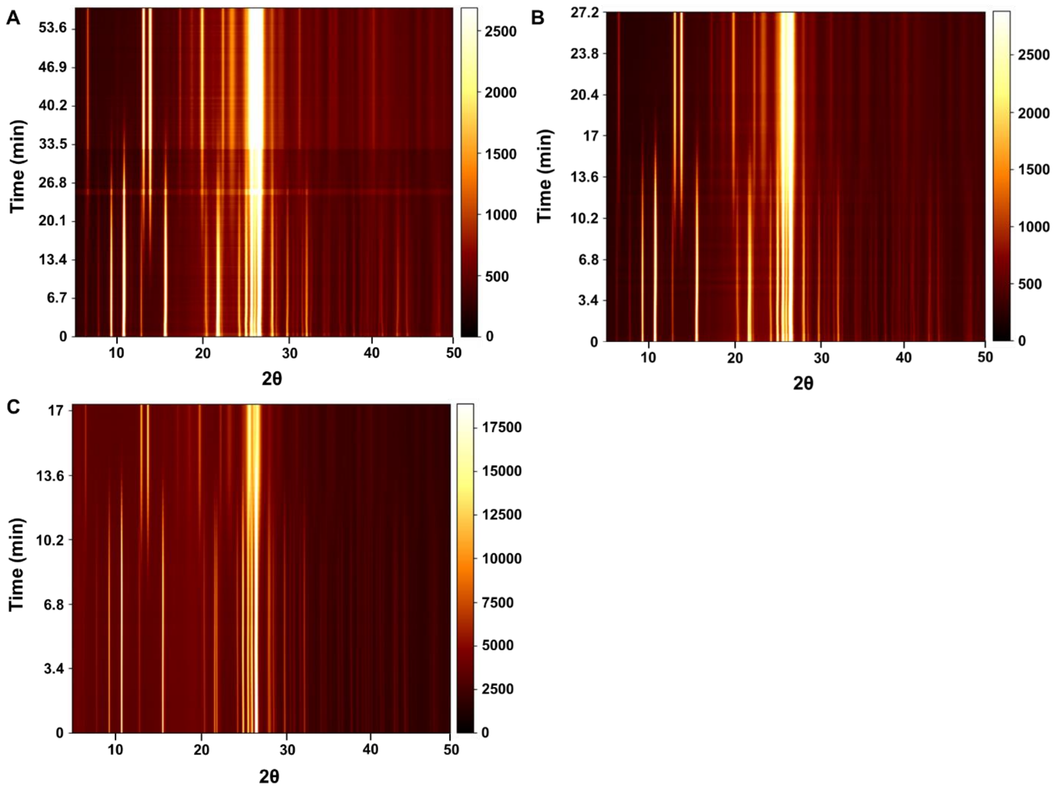

Figure S8. Contour plots of S_{MeOH} heated to and held isothermal at (A) 40, (B) 45, and (C) 50 °C. Samples were either loaded into the capillary as a wet paste (40 and 45 °C) or loaded wet (50 °C). S_{MeOH} was able to completely transition to phase pure Form 1 after approximately 51, 20, and 13 minutes at 40, 45, and 50 °C.

Table S2. Correlation coefficients associated with different solid state reaction models for **S_{MeOH}** (ground) isothermal TGA data (40, 45, and 50 °C). Reaction models with $R^2 > 0.99$ are red and with $R^2 \geq 0.999$ are red and bold.

	**40 °C**	**45 °C**	**50 °C**
**Nucleation Models**			
1D growth of nuclei (Avrami-Erofeyev Eq, n = 2) (**A2**)	0.99726	0.99802	0.99880
2D growth of nuclei (Avrami-Erofeyev Eq, n = 3) (**A3**)	0.99878	0.99675	0.99478
3D growth of nuclei (Avrami-Erofeyev Eq, n = 4) (**A4**)	0.99667	0.99318	0.98971
Random nucleation (Prout-Tompkins Eq) (**B1**)	**0.99923**	0.99760	0.99608
Power law (n = 1/2) (**P2**)	0.97398	0.96592	0.95785
Power law (n = 1/3) (**P3**)	0.96121	0.95080	0.94015
Power law (n = 1/4) (**P4**)	0.95346	0.94186	0.92985
**Geometrical Contraction Models**			
2D phase boundary (Contracting area) (**R2**)	0.99018	0.99397	0.99756
3D phase boundary (Contracting volume) (**R3**)	0.98290	0.98806	0.99303
**Diffusion Models**			
1D diffusion (**D1**)	0.96831	0.97719	0.98552
2D diffusion (**D2**)	0.93997	0.95101	0.96203
3D diffusion (Jander Eq) (**D3**)	0.88517	0.89691	0.90923
3D Diffusion (Ginstling-Brounshtein Eq) (**D4**)	0.92351	0.93502	0.94673
**Reaction Order Models**			
Zero-order (**R1**)	0.99227	0.99098	0.98959
First-order (**F1**)	0.95714	0.96440	0.97159
Second-order (**F2**)	0.80344	0.81097	0.81887
Third-order (**F3**)	0.61759	0.61905	0.62144

A

S_{MeOH} **Desolvation to** F_I	**Ea (kJ/mol)**
**A2** Model	145 ± 26
**A3** Model	144 ± 26
**B1** Model	144 ± 26

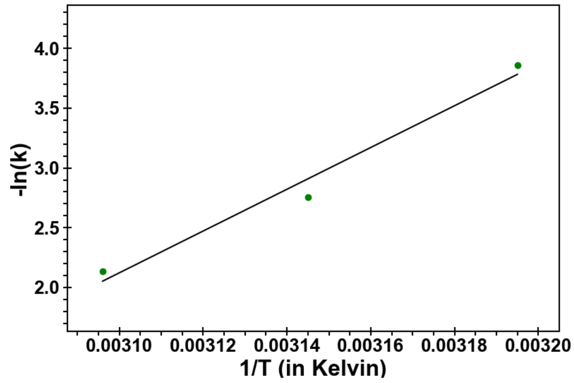

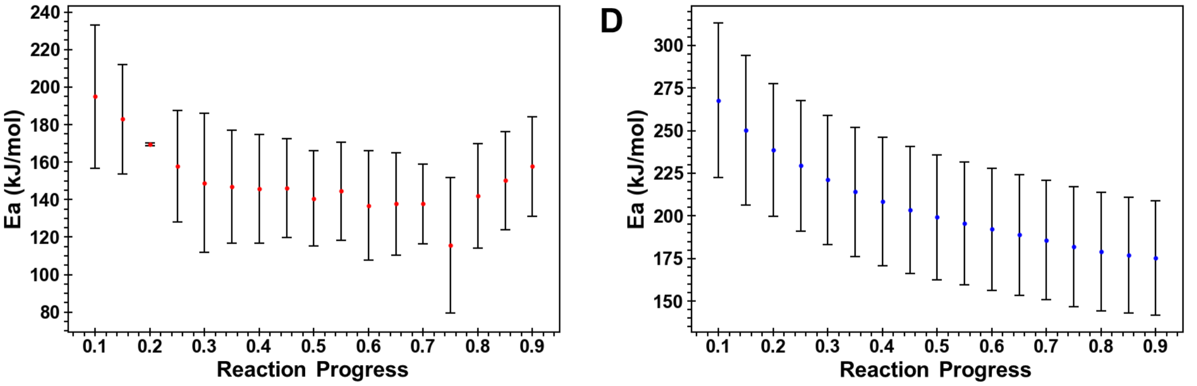

Figure S9. E_a values determined from model-based and model-free kinetic analyses of S_{MeOH} TGA isothermal desolvation at 40, 45 and 50°C. (A) The three nucleation models with the highest R^2 values (A2, A3 and B1) yielded similar E_a values. (B) Representative Arrhenius plot of the A2 model. Time-dependent E_a values calculated from model-free (C) Friedman and (D) Standard methods show a decreasing E_a as a function of reaction progress.

Notes: Friedman Analysis is based on the logarithmic form of the general differential rate law and plotting ln(dα/dt) as a function of 1/T at each α.

$$\ln\left(\frac{d\alpha}{dt}\right)_\alpha = \left(\ln A\, f(\alpha)\right)_\alpha - \frac{E_{a\alpha}}{RT_\alpha}$$

Standard Analysis is based on the logarithmic form of the general integral rate law and plotting -ln(t) as a function of 1/T at each α.

$$-\ln t_\alpha = \ln\left(\frac{A}{g(\alpha)}\right)_\alpha - \frac{E_{a\alpha}}{RT_\alpha}$$

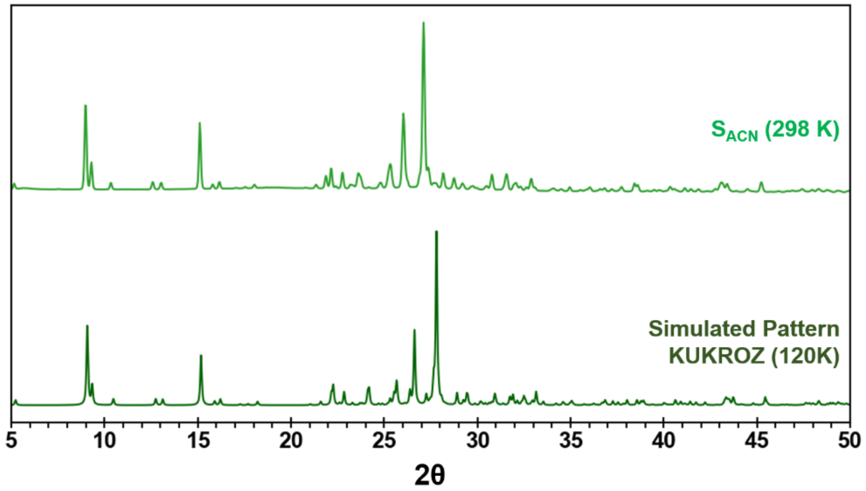

Figure S10. Experimental sPXRD of S_{ACN} at the start of the dehydration experiment compared against the simulated PXRD from the single crystal structure.

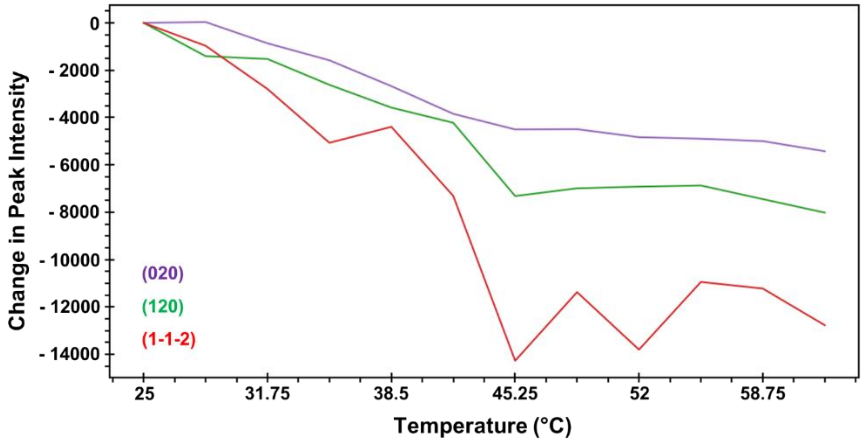

Figure S11. Change in peak intensity for (020), (120), and (1-1-2) diffraction lines of S_{ACN} with temperature. From 25 to 62 °C, the peaks decrease by 18.7%, 24.5%, and 18.6%, respectively.

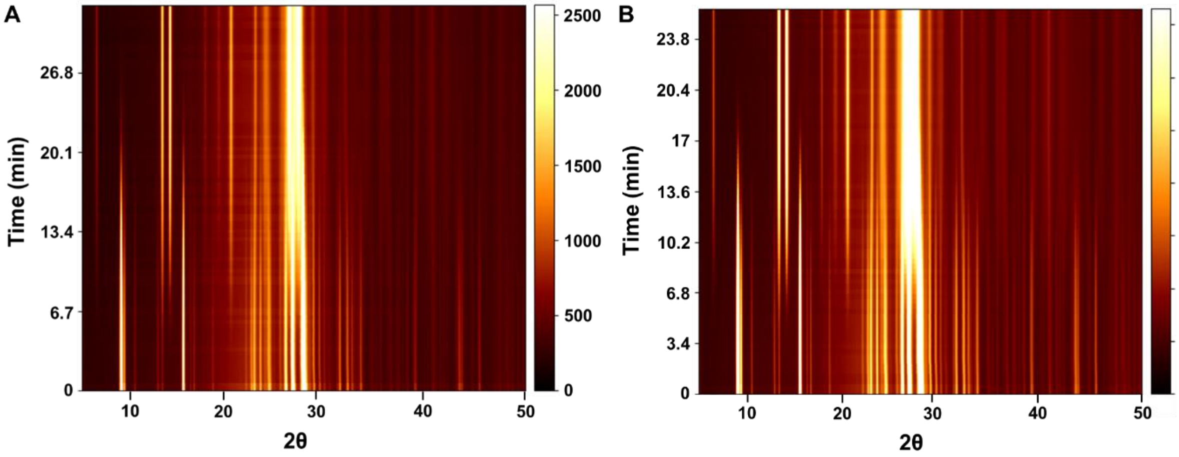

Figure S12. Contour plots of S_{ACN} heated to and held isothermal at (A) 40 °C and (B) 45 °C. Samples were loaded into the capillary as a wet paste (40 and 45 °C) and were able to completely transition to phase pure Form 1 after approximately 31 and 20 minutes, respectively.

Table S3. Correlation coefficients associated with different solid state reaction models for **S_{ACN}** (ground) isothermal TGA data (40, 45, and 50 °C). Reaction models with $R^2 > 0.99$ are red and with $R^2 \geq 0.999$ are red and bold.

	**40 °C**	**45 °C**	**50 °C**
**Nucleation Models**			
1D growth of nuclei (Avrami-Erofeyev Eq, n = 2) (**A2**)	**0.99928**	**0.99902**	0.99891
2D growth of nuclei (Avrami-Erofeyev Eq, n = 3) (**A3**)	0.99702	0.99771	0.99622
3D growth of nuclei (Avrami-Erofeyev Eq, n = 4) (**A4**)	0.99288	0.99404	0.99178
Random nucleation (Prout-Tompkins Eq) (**B1**)	0.99815	0.99862	0.99725
Power law (n = 1/2) (**P2**)	0.96385	0.96705	0.96286
Power law (n = 1/3) (**P3**)	0.94800	0.95176	0.94589
Power law (n = 1/4) (**P4**)	0.93870	0.94273	0.93597
**Geometrical Contraction Models**			
2D phase boundary (Contracting area) (**R2**)	0.99570	0.99485	0.99707
3D phase boundary (Contracting volume) (**R3**)	0.99029	0.98871	0.99127
**Diffusion Models**			
1D diffusion (**D1**)	0.97964	0.97740	0.98330
2D diffusion (**D2**)	0.95415	0.95025	0.95632
3D diffusion (Jander Eq) (**D3**)	0.90045	0.89451	0.89930
3D Diffusion (Ginstling-Brounshtein Eq) (**D4**)	0.93834	0.93374	0.93951
**Reaction Order Models**			
Zero-order (**R1**)	0.99094	0.99228	0.99247
First-order (**F1**)	0.96744	0.96437	0.96714
Second-order (**F2**)	0.81475	0.80711	0.80689
Third-order (**F3**)	0.62262	0.61139	0.60470

A

S_{ACN} Desolvation to F_I	Ea (kJ/mol)
**A2** Model	94.7 ± 26
**A3** Model	94.5 ± 26
**B1** Model	94.5 ± 26

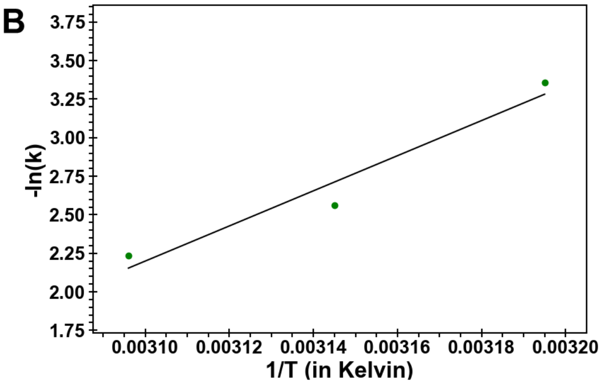

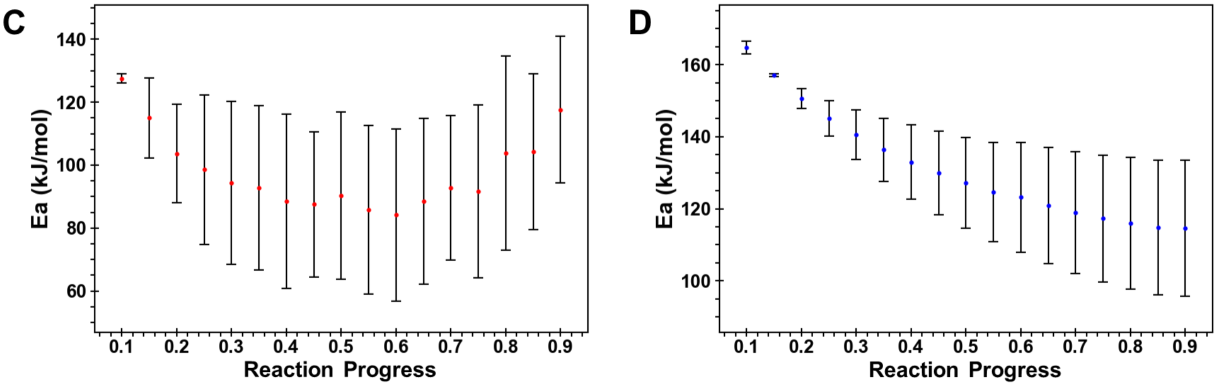

Figure S13. E_a values determined from model-based and model-free kinetic analyses of S_{ACN} TGA isothermal desolvation at 40, 45 and 50°C. (A) The three nucleation models with the highest R^2 values (A2, A3 and B1) yielded similar E_a values. (B) Representative Arrhenius plot of the A2 model. Time-dependent E_a values calculated from model-free (C) Friedman and (D) standard analysis methods.

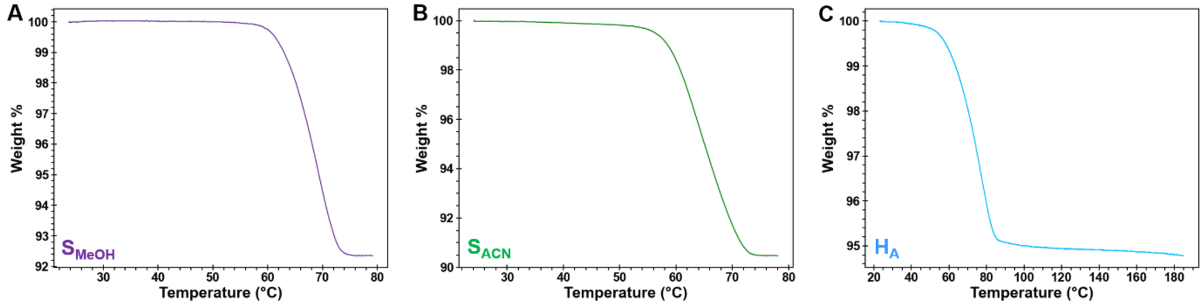

Figure S14. Comparison of TGA of heat flow vs. temperature for (A) S_{MeOH}, (B) S_{ACN} and (C) H_A collected under identical conditions (heating rate = 5 °C/min, open pans).

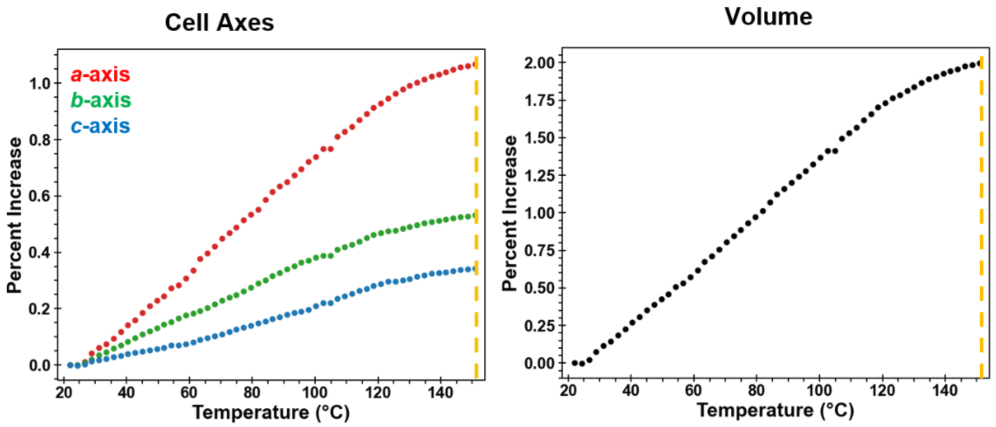

Figure S15. Thermal expansion in H_A and H* over the temperature range up to 150 °C.

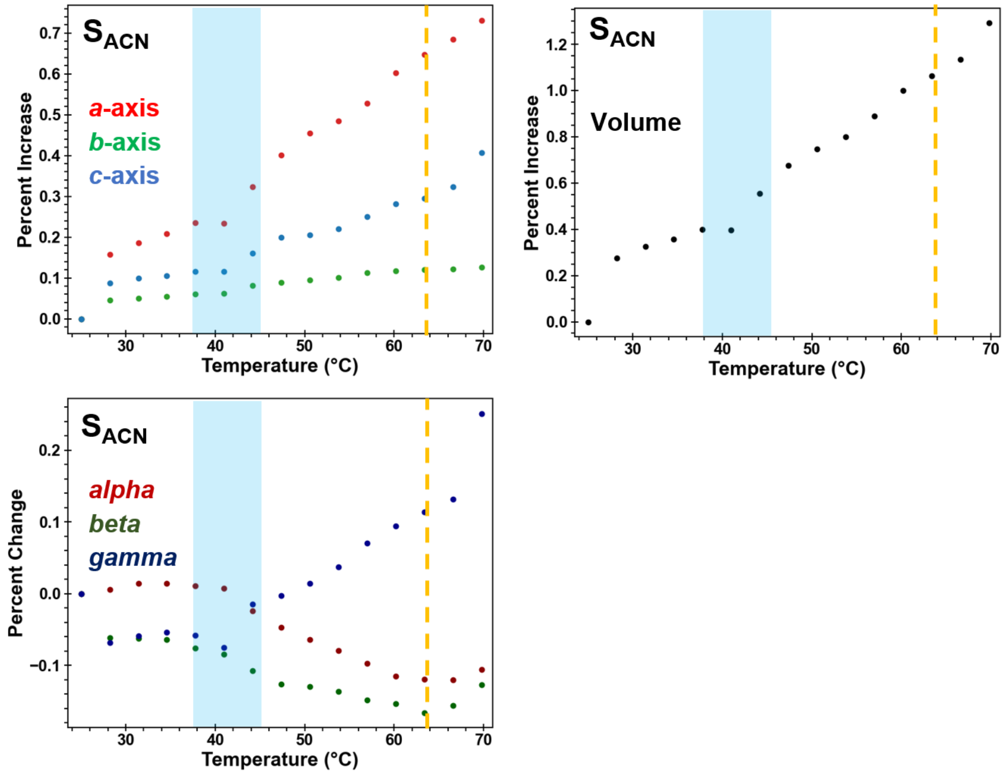

Figure S16. Pawley refined cell parameters for S_{ACN} based on sPXRD desolvation data.

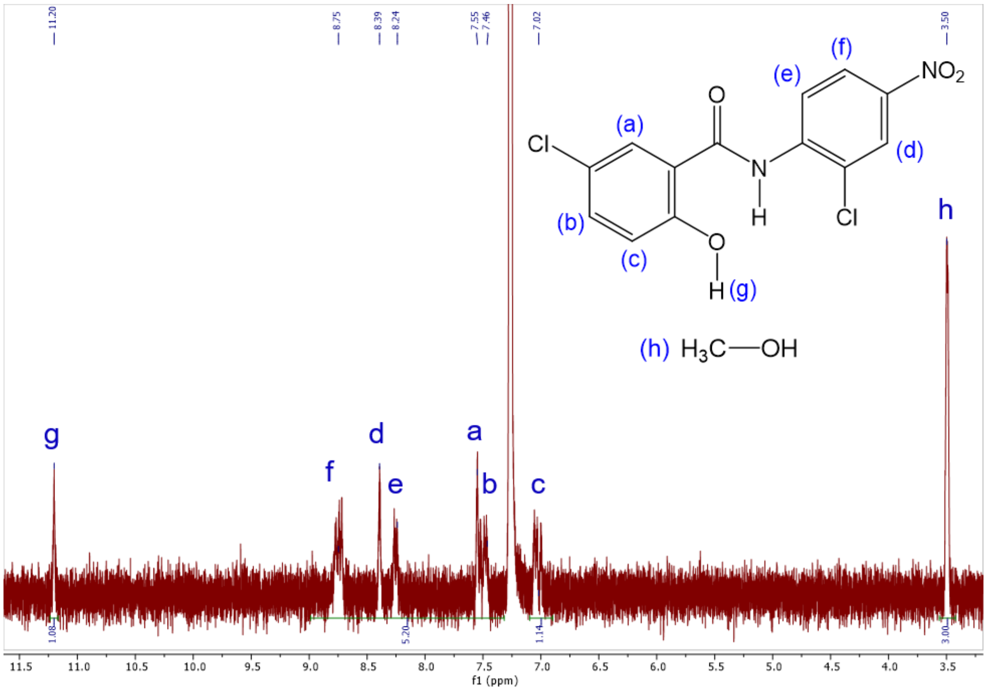

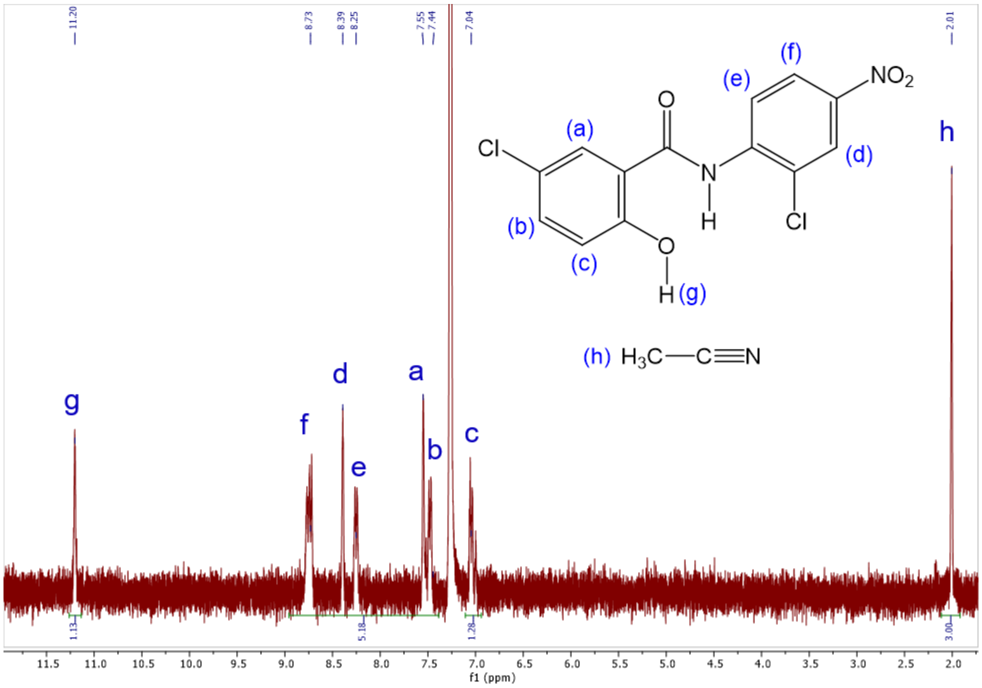

Figure S17. ^{1}H NMR ($CDCl_3$) of dissolved S_{MeOH} and S_{ACN} crystals.
